# Recombinant adenovirus of human p66Shc inhibits MCF-7 cell proliferation

**DOI:** 10.1038/srep31534

**Published:** 2016-08-17

**Authors:** Xiaoshan Yang, Rong Xu, Yajun Lin, Yongzhan Zhen, Jie Wei, Gang Hu, Hongfan Sun

**Affiliations:** 1 Tianjin Key Laboratory of Biomaterial Research, Institute of Biomedical Engineering, Peking Union Medical College & Chinese Academy of Medical Sciences, Tianjin 300192, China; 2 The key Laboratory of Geriatrics, Beijing Hospital &Beijing Institute of Geriatrics, Ministry of Health, Beijing, 100730, China; 3 Department of Histology and Embryology, College of Basic Medical, Hebei United University, Tangshan, 063000, China

## Abstract

The aim of this work was to construct a human recombinant p66Shc adenovirus and to investigate the inhibition of recombinant p66Shc adenovirus on MCF-7 cells. The recombinant adenovirus expression vector was constructed using the Adeno-X Adenoviral System 3. Inhibition of MCF-7 cell proliferation was determined by MTT. Intracellular ROS was measured by DCFH-DA fluorescent probes, and 8-OHdG was detected by ELISA. Cell apoptosis and the cell cycle were assayed by flow cytometry. Western blot were used to observe protein expression. p66Shc expression was upregulated in 4 cell lines after infection. The inhibitory effect of p66Shc recombinant adenovirus on MCF-7 cells was accompanied by enhanced ROS and 8-OHdG. However, no significant differences were observed in the cell apoptosis rate. The ratio of the cell cycle G2/M phase showed a significant increase. Follow-up experiments demonstrated that the expressions of p53, p-p53, cyclin B1 and CDK1 were upregulated with the overexpression of p66Shc. The Adeno-X Adenoviral System 3 can be used to efficiently construct recombinant adenovirus containing p66Shc gene, and the Adeno-X can inhibit the proliferation of MCF-7 cells by inducing cell cycle arrest at the G2/M phase. These results suggested that p66Shc may be a key target for clinical cancer therapy.

p66Shc, a 66 kDa proto-oncogene Src homologous-collagen homologue (Shc) adaptor protein, is an important protein that regulates the levels of reactive oxygen species (ROS) and lifespan in mammals^1^,^2^. Reactive oxygen species are widely accepted as one of the main factors of the aging process. *p66Shc* knockout mice have a lifespan approximately 30% longer and demonstrated an enhanced resistance to oxidative stress^1^ and age-related pathologies, such as atherosclerosis^3^,^4^, endothelial disorders^5^, obesity-induced insulin resistance^6^, AGE (advanced glycation end products)-dependent glomerulopathy related to diabetes mellitus^7^,^8^, and ethanol-induced liver disease^9^. Over the past decade, it was also reported that p66Shc can inhibit cell proliferation though blocking the MAPK or ERK signaling pathways^10^,^11^. Its effect on tumor cells has attracted the attention of researchers. Recently, numerous investigations have demonstrated that p66Shc can also inhibit and kill tumor cells^12^,^13^,^14^. The study of its function and its mechanism of action is particularly important.

At present, researchers have clarified the protective effect of lower levels of p66Shc on the organism mostly using genetic mutation or knockout mutation techniques^15^,^16^,^17^,^18^,^19^. To increase the level of p66Shc expression, only plasmid transfection methods can be utilized to import the exogenous gene into cell lines. Due to the limited gene transfection efficiency, further research of the function of p66Shc, especially in primary cells and *in vivo* studies, is restricted. In this study, we constructed a human p66Shc recombinant adenovirus expression vector (AdenoX-p66Shc) using the Adeno-X Adenoviral System 3, which was easy to use and had a high efficiency for recombinant reactions. We observed a significantly higher expression of p66Shc in AdenoX-p66Shc infected cells, including primary cells, indicating that this tool could potentially be used to research the function of p66Shc *in vitro* and *in vivo* in the future.

## Results

### Construction and expression of human recombinant p66Shc adenovirus

**Figure 1:**
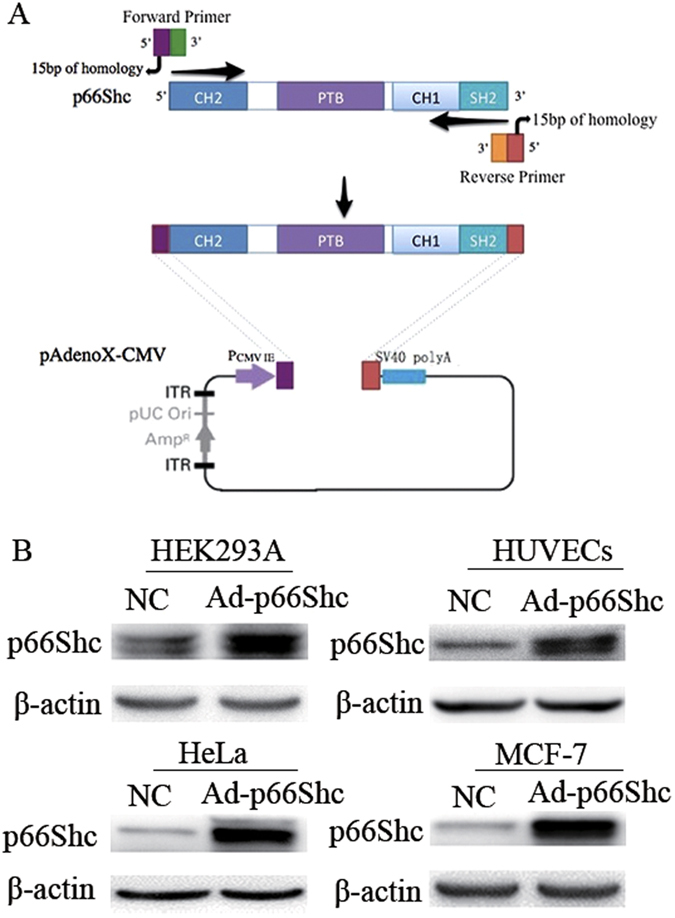
Construction and expression of human recombinant p66Shc adenovirus. (**A**) Schematic presentation of p66Shc homologous recombination with pAdenoX-CMV. CH1: collagen-homology region; PTB: phosphotyrosine-binding domain; SH2: Src-homology2 domain; P_CMV IE_: cytomegalovirus immediate early promoter; SV40 polyA: simian virus 40 polyA signals; ITR: inverted terminal repeat; AMP: ampicillin. (**B**) HEK293A, HUVECs, HeLa and MCF-7 cells were infected with Ad-p66Shc or negative control (NC) for 48 h. The expression of p66Shc protein was detected by Western blot.

The human p66Shc gene was amplified with 15 bp extensions that are homologous to the ends of the linearized adenoviral vector (Fig. 1A). To further validate the efficiency of the recombinant p66Shc adenovirus, HEK293A, HUVECs, HeLa and MCF-7 cells were infected by AdenoX-p66Shc (Ad-p66Shc) or a negative control for 48 h. Western blot analysis revealed that the expression of p66Shc in all of the cells infected by AdenoX-p66Shc was dramatically increased compared with the negative control (Fig. 1B). These results indicated that we had successfully constructed the recombinant adenovirus containing the human p66Shc gene, and the recombinant adenovirus was capable of efficiently infecting different types of cells.

### p66Shc inhibited the proliferation of MCF-7 cells

**Figure 2:**
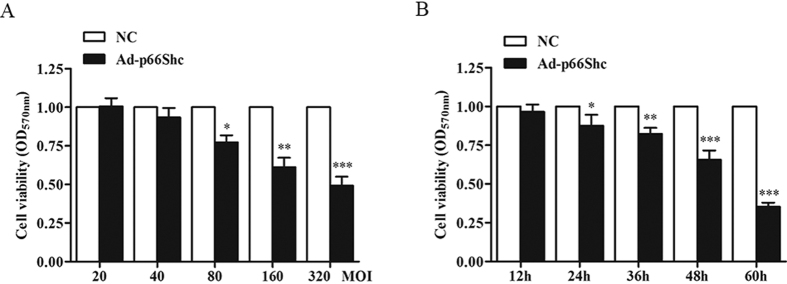
p66Shc inhibited MCF-7 cell viability. (**A**) MCF-7 cells were infected with various concentrations of Ad-p66Shc or negative control (20, 40, 80, 160, or 320 MOI) for 48 h. (**B**) MCF-7 cells were infected with 100 MOI Ad-p66Shc for different amounts of time (12, 24, 36, 48, or 60 h), and cell viability was detected using the MTT method. The data represent the means ± SEM, n=6 independent experiments. *p < 0.05, **p < 0.01, ***p < 0.001 vs NC (NC: negative control).

To determine the effect of p66Shc on cell viability, MCF-7 cells were infected with Ad-p66Shc or a negative control. As shown in Fig. 2A, upregulation of p66Shc by Ad-p66Shc decreased cell viability by 23% at 80 MOI and by 40% at 160 MOI. The cells were cultured for different time (12, 24, 36, 48, 60 h) in the presence of 100 MOI Ad-p66Shc. MCF-7 cells demonstrated decreased cell proliferation over time (Fig. 2B). These results demonstrated that p66Shc could inhibit the proliferation of MCF-7 cells.

### Induction of oxidative stress and associated DNA damage in MCF-7 cells

**Figure 3:**
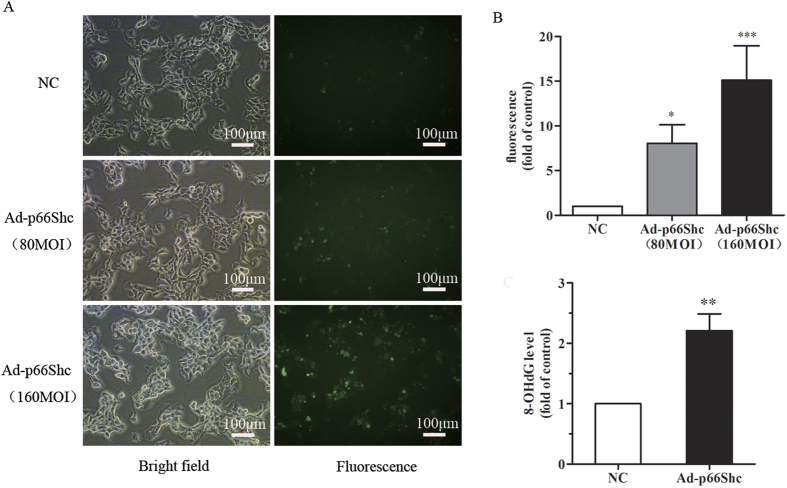
p66Shc increased intracellular ROS, accompanied by an enhanced level of 8-OHdG. (**A**) MCF-7 cells were treated with NC, 80 MOI Ad-p66Shc and 160 MOI Ad-p66Shc for 48 h. DCFH-DA staining showed that p66Shc significantly induced intracellular ROS. *p < 0.05, ***p < 0.001 vs Negative control. (**B**) MCF-7 cells were infected with 100 MOI Ad-p66Shc or negative control for 48 h. The upregulation of p66Shc also increased the level of 8-OHdG. The data represent the means ± SEM, n = 3 independent experiments. **p < 0.01 vs NC (NC: negative control).

Intracellular ROS were stained with DCFH-DA, and the fluorescence in each group was assessed by flow cytometry as described in the Materials and Methods. p66Shc could increase ROS levels in MCF-7 cells in a dose-dependent manner (Fig. 3A), and the DNA oxidative damage product 8-OHdG was significantly increased after Ad-p66Shc infection (Fig. 3B).

### Upregulation of p66Shc induced the expression and phosphorylation of p53

**Figure 4:**
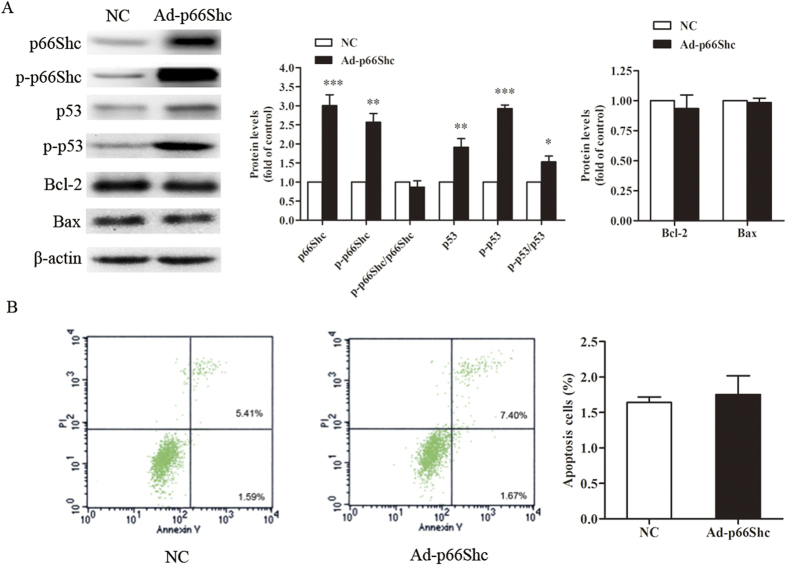
Upregulation of p66Shc induced the expression and phosphorylation of p53. MCF-7 cells were infected with Ad-p66Shc or negative control for 48 h. (**A**) The cell extracts were prepared and analyzed by Western blot with the corresponding antibodies. The quantification of the digital images was performed using ImageJ software. (**B**) MCF-7 cells were stained with annexin V-FITC and PI and analyzed by flow cytometry. The data represent the means ± SEM, n = 3 independent experiments. *p < 0.05, **p < 0.01, ***p < 0.001 vs NC (NC: negative control).

We were also interested in the change of p53 expression and its phosphorylation, which was dependent on our target protein p66Shc, according to our data (Fig. 4A). To determine the mechanism, the levels of proteins related to mitochondrial apoptosis, including Bax and Bcl-2, were examined in MCF-7 cells and showed no significant change (Fig. 4A). The result also shows that the phosphorylation of p66Shc (p-p66Shc) on the S36 residue increased significantly, but the ratio of p-p66Shc to p66Shc was not changed significantly. A flow cytometry assay also verified that the number of apoptotic cells was similar between the cells infected with Ad-p66Shc and the negative control (Fig. 4B). Together, these data indicated that overexpression of p66Shc could increase the level of p53, but mitochondrial apoptosis could not explain the inhibitory effect induced by p66Shc.

### p66Shc induced MCF-7 cell cycle arrest at the G2/M phase

**Figure 5:**
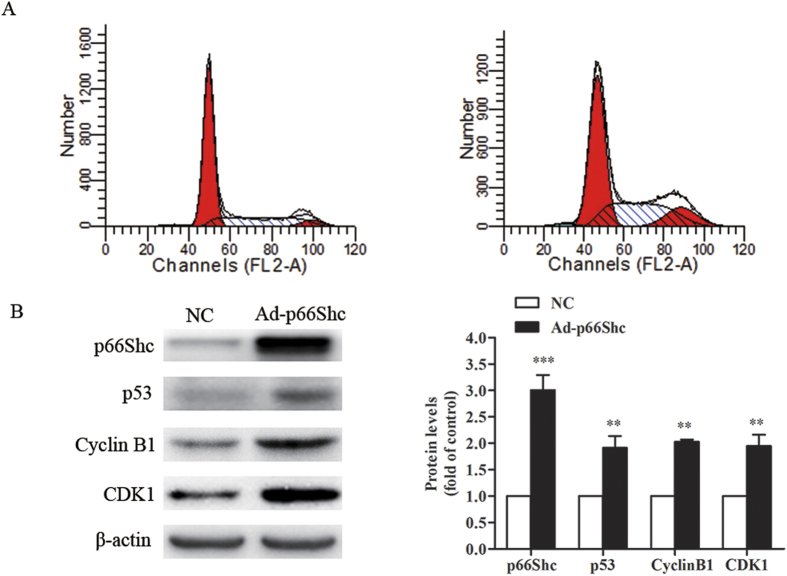
p66Shc induced MCF-7 cell cycle arrest at the G2/M phase. MCF-7 cells were infected with Ad-p66Shc or negative control for 48 h. (**A**) After treatment, the cells were examined by flow cytometer for cell cycle analysis. (**B**) The cell extracts were prepared and analyzed by Western blot with the corresponding antibodies. The quantification of the digital images was performed using ImageJ software. The data represent the means ± SEM, n = 3 independent experiments. **p < 0.01, ***p < 0.001 vs NC (NC: negative control).

To further elucidate the underlying mechanisms responsible for the inhibitory effect of p66Shc on MCF-7 cells, flow cytometry was performed for cell cycle analysis. As shown in Fig. 5A, G2/M arrest was observed in the Ad-p66Shc infected cells compared to the negative control. We also analyzed the protein levels of cyclin B1 and CDK1, which regulate the cell cycle during the G2/M phase. The results revealed that cyclin B1 and CDK1 were dramatically upregulated in Ad-p66Shc infected cells compared to the negative control (Fig. 5B).

## Discussion

Src homology 2 domain containing transforming protein 1 isoform 1, p66Shc, is an important protein, encoded by the Shc A gene, associated with senescence, and it has received extensive attention due to the prolonged life span of the p66Shc^−/−^ mouse^1^,^20^,^21^,^22^. In recent years, the function of p66Shc in promoting ROS production in mitochondria^23^,^24^,^25^,^26^,^27^,^28^ and its role in diseases related to oxidative damage, such as diabetes^8^,^29^,^30^, atherogenesis^3^,^4^, endothelial dysfunction, etc.^5^,^31^, have been investigated in a number of studies.

In the present study, we constructed a human p66Shc recombinant adenovirus expression vector (Adp66Shc) using the Adeno-X Adenoviral System 3. Gene sequencing results and Western blot, indicated that we had successfully constructed the recombinant adenovirus containing our target gene, human *p66Shc*, and the recombinant adenovirus was capable of efficiently infecting different types of cells, including MCF-7 cells (Fig. 1). The results of MTT assay demonstrated that Ad-p66Shc could significantly suppress the proliferation of MCF-7 cells, and the inhibition appears to be dose and time dependent (Fig. 2). We also found that the levels of ROS and 8-OHdG were dramatically increased after infection with Ad-p66Shc (Fig. 3). ROS are natural products generated during cellular metabolism, and 8-OHdG is a biomarker for oxidative DNA damage^32^,^33^,^34^. *p66shc* is a gene that regulates the level of ROS. We conclude that high expression of p66Shc can inhibit MCF-7 cell proliferation by increasing ROS levels and oxidative damage to DNA^4^,^35^,^36^.

The p53 protein, a tumor suppressor gene discovered many years ago, plays a major role in the cellular responses to DNA damage and other genomic aberrations^37^. The activation of p53 can lead to either cell cycle arrest and DNA repair or apoptosis^38^,^39^,^40^. Our previous results revealed an increase in the oxidative DNA damage product 8-OHdG (Fig. 3). Subsequently, we investigated the changes of p53 and phosphorylated p53 (Ser 15). DNA damage induces the phosphorylation of p53 at Ser15 and Ser20 and leads to a reduced interaction between p53 and its negative regulator. Phosphorylation impairs the ability of MDM2 to bind p53, promoting both the accumulation and activation of p53 in response to DNA damage^41^. Our research shows that the upregulation of p66Shc induces the phosphorylation of p53 (Fig. 4A), suggesting that the p53 signaling pathway also participates in the process induced by Ad-p66Shc. Phosphorylation of p66Shc on the S36 residue has been shown to have a pro-apoptotic effect in different cell lines. Our research also shows that the phosphorylation of p66Shc on the S36 residue increased significantly, but the ratio of p-p66Shc to p66Shc was not changed significantly. In addition, we found that the expression of proteins related to apoptosis, including Bax and Bcl-2, were not changed. Meanwhile, the cell apoptosis rate analyzed by flow cytometry did not change (Fig. 4). These results indicated that the activation of p53 did not lead to apoptosis in MCF-7 cells.

The DNA of eukaryotic cells, including vascular cells, is under constant attack by chemicals, free radicals, and ionizing radiation that can be caused by environmental exposure, by-products of intracellular metabolism, or medical therapy^42^,^43^,^44^. These insults eventually result in cell cycle arrest at the G1/S, intra-S, or G2/M checkpoint^45^. The G2/M DNA damage checkpoint serves to prevent the cell from entering mitosis (M-phase) with genomic DNA damage. Specifically, the activity of the cyclin B1-CDK1 complex is pivotal in regulating the G2-phase transition in which CDK1 is maintained in an inactive state^46^. When the DNA repair is successful, the cell cycle arrest may be lifted; if repair fails, permanent cell cycle arrest may occur. Next, we analyzed the cell cycle distribution of MCF-7 cells. The increased number of cells arrested at the G2/M phase is the most likely cause of the inhibitory effect induced by p66Shc (Fig. 5A). Western blot analysis demonstrated an enhanced expression of cyclin B1 and CDK1 followed by an increased level of p53 (Fig. 5B). Cyclin B1 is synthesized in late S and G2 phase, and begins to degrade in metaphase. During other phases, the level of cyclin B1 is very low^46^,^47^. This finding suggests that the high expression of p66Shc caused MCF-7 cell cycle arrest at the G2/M phase, preventing the cells from dividing into two daughter cells, which is also consistent with the results of the flow cytometry analysis (Fig. 5A).

In conclusion, these results suggested that AdenoX-p66Shc inhibited MCF-7 cell proliferation and induced cell cycle arrest at the G2/M phase by increasing the ROS level and the expression of p53. A further understanding of the biological function of p66Shc is of great importance, as p66Shc may serve as a potential drug target to treat age-related diseases and cancer.

## Materials and Methods

### Reagents and antibodies

The reagents 3-(4,5-dimethylthiazol-2-yl)-2,5-diphenyl-tetrazolium bromide (MTT) and dimethyl sulfoxide (DMSO) were obtained from Sigma Aldrich (St. Louis, MO, USA). Antibodies against p53, p-p53, Bax, Bcl-2, cyclin B1, CDK1 and β-actin were purchased from Cell Signaling Technology (Beverly, MA, USA). An antibody against p66Shc, p-p66Shc (S36) was purchased from BD Biosciences (Franklin Lakes, NJ, USA). Secondary antibodies were purchased from Cell Signaling Technology.

### Cell culture

MCF-7 cells, HEK293A cells and HeLa cells were cultured in Dulbecco’s Modified Eagle’s Medium (DMEM) supplemented with 10% fetal bovine serum (Hyclone, Logan, UT, USA), 100 units/ml penicillin and 100 μg/ml streptomycin (Invitrogen Corporation, Carlsbad, CA, USA) at 37 °C in a humidified atmosphere of 5% CO_2_.

Human umbilical vein endothelial cells (HUVECs) were isolated and cultured in M199 medium supplemented with 20% fetal bovine serum (Hyclone, Logan, UT, USA), 2 mM glutamine, 100 units/ml penicillin and 100 μg/ml streptomycin (Invitrogen Corporation, Carlsbad, CA, USA) at 37 °C in a humidified atmosphere of 5% CO_2_. Human umbilical vascular endothelial cells at passages 2–4 were used for this study.

### Construction and expression of human recombinant p66Shc adenovirus

The human p66Shc gene was amplified by PCR using pcDNA3.1 his-p66Shc (Addgene, Cambridge, MA, USA) and was directly cloned into a linearized adenoviral vector (Adeno-X Adenoviral System 3 (CMV) kit) *in vitro*. After confirmation using DNA electrophoresis and gene sequencing, the linearized recombinant plasmid was transfected into HEK293A cells for packaging. HEK293A, HUVECs, HeLa and MCF-7 cells infected by AdenoX-p66Shc (Ad-p66Shc) were used to validate the efficiency of infection. AdenoX-LacZ (Ad-LacZ) provided by the kit was used as a negative control (NC). The p66Shc expression in each cell line was detected by Western blot.

### Dimethyl thiazolyl diphenyl tetrazolium (MTT) assay

To explore the effect of Ad-p66Shc, 4 × 10^3^ cells per well in 100 μl medium were seeded in 96-well plates and infected with Ad-p66Shc or negative control (NC). Twenty microliters of the 3-(4,5-dimethylthiazol-2-yl)-2,5-diphenyltetrazolium bromide (MTT) reagent (Sigma, St. Louis, MO, USA) was added to the wells and incubated with the cells for 4 h. After removing the medium, blue formazan was dissolved with 150 μl dimethyl sulfoxide (DMSO), and the absorbance was measured at 570 nm. Wells containing only MCF-7 cells served as blanks.

### ROS detection

MCF-7 cells (1 × 10^5^ cells/ml) were treated with negative control (NC) or Ad-p66Shc for 48 h and then labeled with 10 μmol/L DCFH-DA (Sigma, USA) for 45 min. ROS generation was indicated by green florescence and was visualized using a fluorescence microscope (Tokyo, Japan). The fluorescence in each group was assessed by flow cytometry with an excitation wavelength of 488 nm and an emission wavelength of 525 nm.

### Enzymatic assessment of Human-8-Hydroxy-2’-desoxyguanosine (8-OHdG)

The level of 8-OHdG in MCF-7 cells was detected by an enzyme linked immunosorbent assay (ELISA) kit (Beijing Keyingmei Technology Co., Ltd) according to the instructions of the manufacturer. In brief, 100 μl of cell lysate was added to an antibody pre-coated microtiter plate and was incubated with 50 μl of conjugate at 37 °C for 1 h before adding 8-OHdG’s substrate. After a 15-min incubation at room temperature, a 50 μl stop solution was added, and the absorbance was assessed at 450 nm.

### Apoptosis assay

The percentage of cells that underwent apoptosis or necrosis was determined using the Annexin V-FITC and propidium iodide (PI) analysis kit (Bio-vision, USA). Forty-eight hours after treatment with Ad-p66Shc (100 MOI) or a negative control (NC), MCF-7 cells were harvested in a 5 ml tube. Then, the cells were washed with cold PBS and resuspended at a final concentration of 1 × 10^6^ cells/ml. Annexin V-FITC (5 μl) and PI (5 μl) were gently mixed and incubated with the cells for 15 min at room temperature. After incubation, the samples were analyzed by flow cytometry within 1 h.

### Flow cytometry for cell cycle analysis

The cells treated with Ad-p66Shc (100 MOI) or a negative control (NC) were harvested, permeabilized, and fixed in 70% ethanol overnight. Prior to the staining, the cells were treated with RNase A to remove RNAs from the cells. Then, PI staining and flow cytometry were performed for cell cycle analysis.

### Western blot

Proteins were extracted from MCF-7 cells using RIPA buffer (Solarbio, China) supplemented with a protease inhibitor cocktail (Cell Signaling Technology). The cell lysates were separated by 12% SDS-PAGE and electrophoretically transferred onto PVDF membranes. The membranes were blocked with 8% milk for 2 h and incubated with specific primary antibodies overnight. After 5 washes in TBST (TBS containing 0.1% Tween 20), the membranes were incubated with HRP-conjugated secondary antibodies in TBST for 2 h. Each membrane was developed using an enhanced ChemiImager 5500 chemiluminescence system (Alpha Innotech Corporation, Miami, FL, USA). β-actin was used as the internal control. The quantification of the digital images was performed using ImageJ software.

### Statistical analysis

All of the statistical calculations were performed using GraphPad Prism 5 software. The data are expressed as the mean ± SEM. Student’s *t*-test was used to compare two conditions, and one-way ANOVA with Bonferroni correction was used for multiple comparisons. P values less than 0.05 were considered significant.

## Additional Information

**How to cite this article**: Yang, X. *et al*. Recombinant adenovirus of human p66Shc inhibits MCF-7 cell proliferation. *Sci. Rep.* **6**, 31534; doi: 10.1038/srep31534 (2016).
